# A novel method for mapping village-scale outdoor resting microhabitats of the primary African malaria vector, *Anopheles gambiae*

**DOI:** 10.1186/s12936-016-1534-9

**Published:** 2016-09-22

**Authors:** Julius R. Dewald, Douglas O. Fuller, Günter C. Müller, John C. Beier

**Affiliations:** 1 Department of Geography and Regional Studies, University of Miami, Coral Gables, FL USA; 2 Kuvin Center for the Study of Tropical and Infectious Diseases, Hadassah Medical School, Hebrew University, Jerusalem, Israel; 3 Department of Public Health Sciences, Miller School of Medicine, University of Miami, Miami, FL USA

**Keywords:** Species distribution modeling, Anopheles, Dempster-Schafer modeling, Mali, Resting habitats

## Abstract

**Background:**

Knowledge of *Anopheles* resting habitats is needed to advance outdoor malaria vector control. This study presents a technique to map locations of resting habitats using high-resolution satellite imagery (world view 2) and probabilistic Dempster-Shafer (D-S) modelling, focused on a rural village in southern Mali, West Africa where field sampling was conducted to determine outdoor habitat preferences of *Anopheles gambiae*, the main vector in the study area.

**Methods:**

A combination of supervised and manual image classification was used to derive an accurate land-cover map from the satellite image that provided classes (i.e., photosynthetically active vegetation, water bodies, wetlands, and buildings) suitable for habitat assessment. Linear fuzzy functions were applied to the different image classes to scale resting habitat covariates into a common data range (0–1) with fuzzy breakpoints parameterized experimentally through comparison with mosquito outdoor resting data. Fuzzy layers were entered into a Dempster-Shafer (D-S) weight-of-evidence model that produced pixel-based probability of resting habitat locations.

**Results:**

The D-S model provided a highly detailed suitability map of resting locations. The results indicated a significant difference (p < 0.001) between D-S values at locations positive for *An. gambiae* and a set of randomly sampled points. Further, a negative binomial regression indicated that although the D-S estimates did not predict abundance (p > 0.05) subsequent analysis suggested that the D-S modelling approach may provide a reasonable estimate locations of low-to-medium *An. gambiae* density. These results suggest that that D-S modelling performed well in identifying presence points and specifically resting habitats.

**Conclusion:**

The use of a D-S modelling framework for predicting the outdoor resting habitat locations provided novel information on this little-known aspect of anopheline ecology. The technique used here may be applied more broadly at different geographic scales using Google Earth, Landsat or other remotely-sensed imagery to assess the malaria vector resting habitats where outdoor control measures can reduce the burden of the disease in Africa and elsewhere.

## Background

Malaria remains one of the most serious public health problems in the developing world, and is considered a high priority for control and elimination within endemic regions [1–4]. Programmes such as the Roll Back Malaria (RBM) have helped reduce malaria by 47 % between 2000 and 2013 globally and by 54 % in the World Health Organization (WHO) African Region [5]. Further, the WHO has set new goals for global malaria reduction by 2030, which include the reduction of global malaria incidence and mortality rates by at least 90 %, as well as elimination of the disease in at least 35 endemic countries [6]. In West Africa, the disease remains particularly problematic, with 15 out of 18 West African countries having the highest malaria transmission rates on the continent.

The spatially extensive nature of malaria in Africa can be explained by the ecology and behaviour of a highly competent vectorial system of *Anopheles* mosquitoes, primarily *Anopheles gambiae*, *Anopheles arabiensis* of the *An. gambiae* complex, and *Anopheles funestus* [7]. Complicating control strategies, these vector species often display different habitat preferences and life histories. For example, adult *An. gambiae* and *An. funestus* feed frequently and predominantly on humans, rest mainly inside houses (i.e., endophily), and can survive for relatively longer periods (39–44 days) relative to adult *An. arabiensis*, which typically survive between 11 and 17 days [8, 9]. Peak densities of *An. gambiae* and *An. arabiensis* follow seasonal patterns of rainfall and both use a range of freshwater larval habitats to breed [10]. *An. funestus* typically proliferates in permanent swamps and reaches peak densities after seasonal rains into the dry season [10]. *An. arabiensis,* which has the most extensive geographic range in of all dominant vectors in sub-Saharan Africa, typically favours more arid habitats relative to the other dominant African vectors [11]. This species has proven difficult to control because of its outdoor (exophilic) resting and combined anthropophilic and zoophilic feeding behaviours [12, 13]. This study focused only on *An. gambiae*, which was the most common species in the study area. Although studies have shown that *An. gambiae* is primarily endophilic [14–17], other studies have also shown that adult *An. gambiae* females also prefer outdoor environments [18–21]. The ability of *An. gambiae* to survive indoors as well as outdoor environments reveals a gap in the understanding of this species’ ecology.

In recent years, much of the effort to control anophelines has focused on development of outdoor strategies that target larvae [22–24]. Relatively novel methods such as attractive toxic sugar baits (ATSB) provide effective means to control adult mosquito populations within and around settlements where mosquitoes typically rest. The use of ATSB, for example, has been shown to decrease male and female *An. gambiae* populations by 90 % and eliminate most older females [25, 26].

Efficient use of these adult targeted outdoor vector control strategies largely depends on knowledge of where the adult mosquitoes are within the environment surrounding communities at risk. Resting sites of adult mosquitoes in general are poorly documented in the literature, despite their importance in the life cycles of these organisms [27–29]. Resting habitats for mosquitoes are defined as areas where mosquitoes remain after emergence, after taking blood meals and/or before oviposition or during periods of inactivity during the daylight hours. Identification of high-probability resting habitats can advance vector control in rural areas by leading to more efficient targeting of vector control interventions around rural villages and large settlements [30]. Thus, highly detailed maps that display the location of resting habitats can provide a key tool to guide outdoor control efforts, particularly placement of ATSB bait stations, insecticide spraying, and larvicides that reduce mosquito populations.

As small poikilothermic organisms, mosquitoes [31] seek shaded areas for the majority of the day to avoid severe desiccation and heat stress that may result in direct sun in tropical locations [32]. This suggests that the daytime resting habitats for these organisms are usually associated with dense vegetation, which provides shade and thus a cooler microclimate relative to open areas such as bare soil, agricultural fields, and built-up spaces. Furthermore, proximity to water bodies and to blood meals provides important environmental resources for Anophelines, as they require shallow, temporary bodies of fresh water that may contain floating and submerged algae, emergent grass, or rice to deposit their eggs [11, 33–39]. The proximity to water is thus considered an important factor when identifying resting habitats [40–43].

Various methods exist to map habitat suitability and presence of mosquitoes from point observations, including Maxent [44] and genetic algorithms [45]. However, these methods are often employed in conjunction with multiple bioclimatic and topographic covariates to map probable presence over large regions or at continental scales. Moreover, to map habitat preferences at local (i.e., village) scales at high resolution (<10 m), different methods may be required in developing countries where high-resolution climate and topographic data are often unavailable at the local scales and spatial resolutions needed to accurately guide vector control activities. However, land cover maps derived from multispectral satellite imagery can provide a range of potential covariates that are specific to mosquito habitat preferences. This study employs Dempster-Shafer (D-S) weight-of-evidence modelling, a variant of Bayesian Theory to generate probability surfaces of habitat suitability. Unlike the Bayesian Theory, the D-S approach does not assume that one has full information and thus D-S models work well when one has incomplete knowledge that changes over time [46, 47] as validation data and other information become available. As the results are based on a small set of land cover classes derived from multispectral imagery, this study demonstrates the feasibility of modelling probability of vector presence and potentially abundance using a more limited set of covariates than are typically employed in other species distribution models.

## Methods

### Study area

**Fig. 1:**
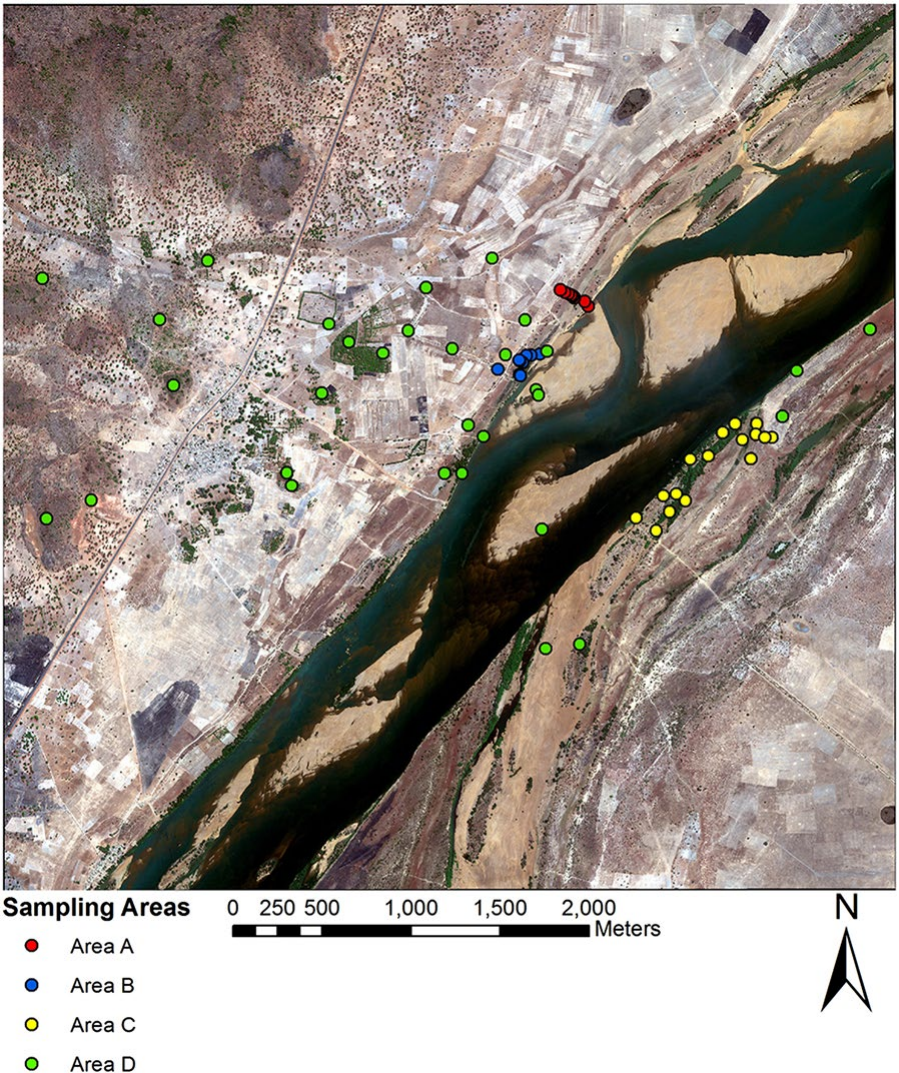
Individual *points* mark the locations of the sampling locations within the study site and the *colour* detonates the area which each location is associated with

The study area was centered around the village of Kenieroba (see Fig. 1), which is located 71 km southwest of Bamako, Mali. The study area covers approximately a 25 square km surrounding the village, which contained approximately 1000 houses as visually estimated from a high resolution WorldView 2 image of the area. A floodplain of about 2 km wide separates Keineroba from the Niger River. During the rainy season (June–September) villagers use parts of the floodplain for rice agriculture, which may be used by vectors as larval habitat. This study site was selected due to its mixture of various land covers, which include a close proximity to the Niger River, dense agricultural plots and sparse natural vegetation, along with areas of human occupation. Focus was placed on the prediction of resting habitats during the early dry season when adult vector densities are generally low relative to the wet season. During the dry season larval habitats become limited as the surface area of water bodies decreases owing to evaporation, whereas these habitats increase sharply at the onset of the rainy season and become abundant and extensive where micro-depressions are found in the landscape [48]. Focusing on mosquito control efforts during the dry season, when vector populations are typically limited by low water availability, may provide a more effective way to control local mosquito populations relative to control efforts during the wet season [49].

### Field data

**Fig. 2:**
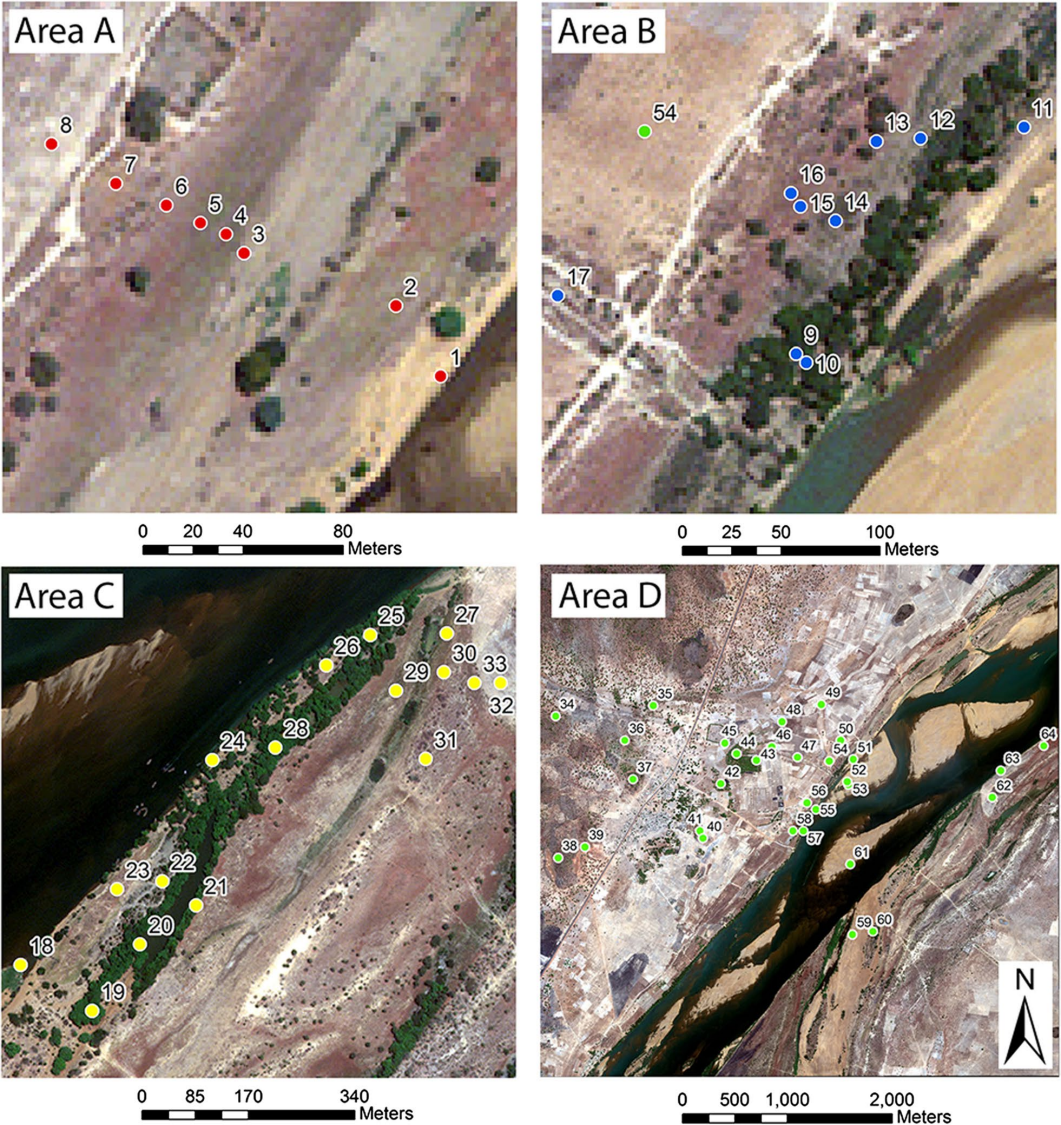
Detailed images showing sampling areas and exact locations where drop nets were placed. The numbers refer to the ID given to each sampling location and correspond with the ID values found in Table 1

Field teams collected adult mosquitoes using drop-net collection methods during November and December 2013. The drop-net captured adult mosquitoes resting in grasses and other herbaceous vegetation near woody canopies as well as in open areas that were not underneath any trees or shrubs. Each drop-net enclosed a 2 × 2 m area and was deployed at predetermined microhabitat locations representing a variety of outdoor habitats. Each drop-net contained a suspended Center for Disease Control light trap (CDC-UV light trap) to capture the resting mosquitoes in the enclosed space within the net. The locations of each sampled location can be seen in Fig. 1, and close up views can be seen in Fig. 2. Study area A was situated next to an existing wetland area by the River Niger, and specifically the sampling locations in this area radiated out from a temporary lagoon. Sampling areas B and C were located in and around riverine forests at opposite banks of the Niger River. The remaining sampling locations were placed at points of interest across the study area and have been grouped together as Area D. These points of interest include mango plantations, open agricultural fields, sandy islands, and various savanna habitats. Sampling in each microhabitat type was done during a 2-to-3 day period and a total of 36 drop-nets were employed in each sampling location with a total of 18 placed in mornings and 18 in the afternoons. After 12 h, the traps were removed and the Anophelines were counted for each site to provide an estimate of abundance at each microhabitat location (see Table 1). Furthermore, the mosquito species collected were identified using PCR (polymerase chain reaction) [50]. Only *An. gambiae* were included in this study. The locations for these microhabitat sampling locations are shown in Fig. 2. These sampling locations were chosen using a non-random sampling design that aimed to assess different habitats where anopheline mosquitos were likely to be found. To assess spatial autocorrelation in the field observations, a Moran’s I statistic was applied to the abundance data.

**Table 1:** Field data

ID	Area	Mosquito sum	Sex: F/M	ID	Area	Mosquito sum	Sex: F/M	ID	Area	Mosquito sum	Sex: F/M
1	A	0	0/0	23	C	1	1/0	45	D	0	0/0
2*	A	3	2/2	24	C	2	2/0	46	D	0	0/0
3	A	115	55/60	25*	C	323	241/82	47*	D	0	0/0
4*	A	58	38/20	26	C	6	5/1	48	D	0	0/0
5	A	188	113/75	27	C	128	104/24	49	D	0	0/0
6*	A	38	23/15	28	C	201	143/58	50	D	0	0/0
7	A	1	1/0	29	C	182	143/39	51	D	0	0/0
8	A	0	0/0	30	C	21	15/6	52	D	0	0/0
9	B	4	3/1	31	C	2	2/0	53	D	0	0/0
10	B	33	23/10	32*	C	0	0/0	54	D	0	0/0
11	B	215	164/51	33	C	7	6/1	55*	D	0	0/0
12	B	113	89/24	34	D	0	0/0	56*	D	0	0/0
13*	B	19	16/3	35	D	0	0/0	57	D	0	0/0
14	B	44	36/8	36*	D	0	0/0	58	D	0	0/0
15	B	1	1/0	37*	D	0	0/0	59*	D	0	0/0
16	B	4	4/0	38	D	0	0/0	60	D	0	0/0
17	B	209	143/66	39	D	0	0/0	61*	D	0	0/0
18	C	1	1/0	40	D	0	0/0	62	D	0	0/0
19*	C	3	2/1	41*	D	0	0/0	63*	D	0	0/0
20	C	14	11/3	42	D	0	0/0	64	D	0	0/0
21	C	116	96/20	43	D	0	0/0				
22	C	138	112/26	44	D	0	0/0				

Pearson Correlation Coefficient of male to female resting site preference: 0.916

* Points that were excluded from the D-S model training and reserved for testing

### Remotely-sensed imagery

A cloud-free, high-resolution WorldView 2 satellite image from March 5, 2013 (2.0 m spatial resolution) was used to map land cover around the study site. The imagery contained four multispectral bands covering the red (0.63–0.69 μm), green (0.51–0.58 μm), blue (0.45–0.51 μm), and near infrared (0.705–0.745 μm) wavelengths. Although image archives did not yield cloud-free WorldView 2 images coincident with the field sampling, the imagery covered the same dry season during which field collections occurred. A classified map was generated from the WorldView 2 imagery using a combination of supervised classification and an image segmentation algorithm to identify discrete objects such as clumps of dense woody vegetation, metal-roofed buildings, water bodies, open fields, and bare earth. Owing to spectral similarity, it was not possible to accurately separate woody vegetation and photosynthetically active grass canopies. However, most areas classified as dense vegetation were associated with tree crowns as the majority of grasses had senesced during this time of the year. A segmentation algorithm was applied to improve classification results as this approach often outperforms per-pixel classification approaches [51, 52]. The segmentation merged pixels into non-overlapping homogeneous objects, which was then used to assign land cover groups for a classification method [53]. An additional ‘wetlands’ class was also included in the final classified image by identifying low-lying areas of areas of alluvial soils that were inundated during field sampling.

### Classification accuracy

Accuracy assessment of the classified image was done using a colour composite image and 280 random points created in ArcMap. The points were randomly stratified across the different classes and care was taken to ensure that the points used for accuracy assessment and training were mutually exclusive. A nearest neighbour analysis was employed to confirm that the points were randomly distributed. The wetlands class was not included in the classification accuracy assessment as it was created independent of the classification algorithms. To further increase class accuracy, a modal filter with a 3 × 3 window was used to eliminate isolated, misclassified pixels. Obviously misclassified features were corrected manually using Google Earth imagery as a cross-reference. Specifically, certain shallow water bodies were classified as metal-roofed buildings by the segmentation algorithm due to the spectral similarity across the four bands. It is also important to note that only buildings with metal roofs were identified in the classified map. These structures comprised approximately 45 % of the total buildings, with the remainder having roofs constructed from thatch materials that could not be distinguished spectrally from senesced vegetation. Furthermore, the size of these buildings was very small, the majority of the buildings being only 3–5 m in diameter.

### D-S modelling

Two hypotheses were used to construct a framework for D-S modelling of resting habitat probability. Firstly, as vegetation can provide substantial amounts of shade, there was an assumption that resting habitats are associated with areas consisting of savanna trees, shrubs, and grasses. Further, the hypothesis was formulated based on the assumption that resting areas are found close to water and that such locations will likely contain higher densities of resting mosquitoes than areas distant (e.g., >1 km) from water bodies. While proximity to the potential blood meals should also influence resting site selection, this hypothesis was excluded from the D-S modelling framework as no outdoor-resting anopheles mosquitoes were found in any of the sampling locations near human habitations.

The classes of dense vegetation, open water, and wetlands were extracted from the classified image and transformed into distance layers using the distance module in IDRISI GIS software [46]. The distance module calculates the distance of each pixel in the study area to the nearest set of target pixels (e.g. vegetation, open water, or wetlands). These distance maps were then converted into fuzzy classes that were scaled from 0 to 1 representing the degree of class membership [54]. A key consideration was the establishment of breakpoints and function selection for fuzzy set membership with breakpoints established where membership began to fall below 1 (breakpoint C) to where membership becomes 0 (breakpoint D). The different fuzzy functions (sigmoidal, J-shaped curve, and user-defined linear) determined the shape of the transition from full to zero class membership (i.e., 1–0).

**Fig. 3:**
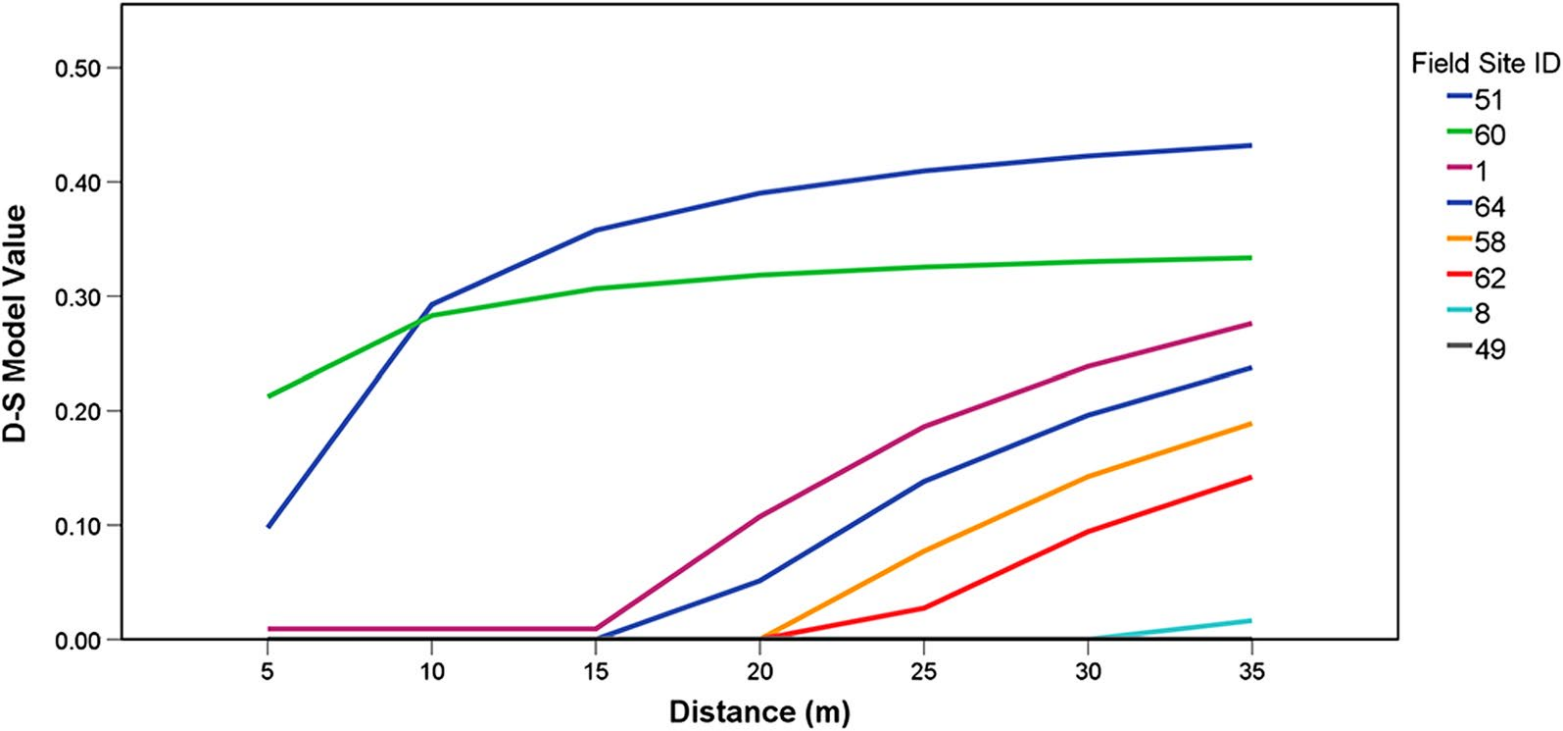
Changes in D-S model values with changing distance-from-vegetation breakpoint values (no count values). The distance at which the D-S value rose above zero for all the affected sites were averaged together for the final D-break point value. The field site ID correlates with the ID values found in Table 1

48 field sample sites (75 %) were randomly selected for in the creation of the D-S model (see Table 1). To create the D-S model, each parameter was entered into the Belief module in the IDRISI GIS software with an indication of their supported hypothesis (resting habitat or not). The module then accumulated evidence in support of each hypothesis by calculating a state of knowledge using the Dempster-Shafer rule of combination, from which a belief image representing the total support for the hypothesis was produced. Multiple iterations of D-S models were created to adjust the fuzzy breakpoint values. The D-S probability values of multiple D-S model iterations were plotted against distance for sample sites that had zero mosquito counts. Each plot revealed how changes in the breakpoint values affect the D-S probability values for each land cover while the other variables are kept constant. The distance at which the probability value began to rise above zero (p > 0) was plotted and as a way to determine an appropriate breakpoint for each fuzzy function included in the D-S model and the mean distance where the condition p > 0 was true for each set of locations with zero mosquito counts was used to establish the final breakpoint value. In this way, commission errors were limited to zero. In the case of curves representing distances from vegetation (n = 6), water (n = 8), and wetland (n = 4), the mean p > 0 values were 17.5 (sd = 8.22), 568.75 (sd = 402.61), and 77.5 (sd = 20.21) respectively. These distances were then used as the final D-S break points values for the fuzzy layers. Figure 3 provides a graphical example of how breakpoint determination was done using this approach.

As a way to validate the results, a *t* test was performed for independent samples (assuming unequal variances) to compare D-S values for the 30 locations that were positive for *An. gambiae* with a sample of 50 random points. Further, since the field data provided mosquito counts in different locations, a negative binomial model (SPSS Version 22, IBM Corp. 2013. Armonk, NY: IBM Corp) was used to assess whether the D-S model may be related to abundance of *An. gambiae* in the landscape. In so doing, the assumption was made that D-S probability should be positively related to mosquito abundance. The negative binomial regression used the remaining 25 % (16 locations) of sampling locations that were omitted from the D-S model.

## Result*s*

**Fig. 4:**
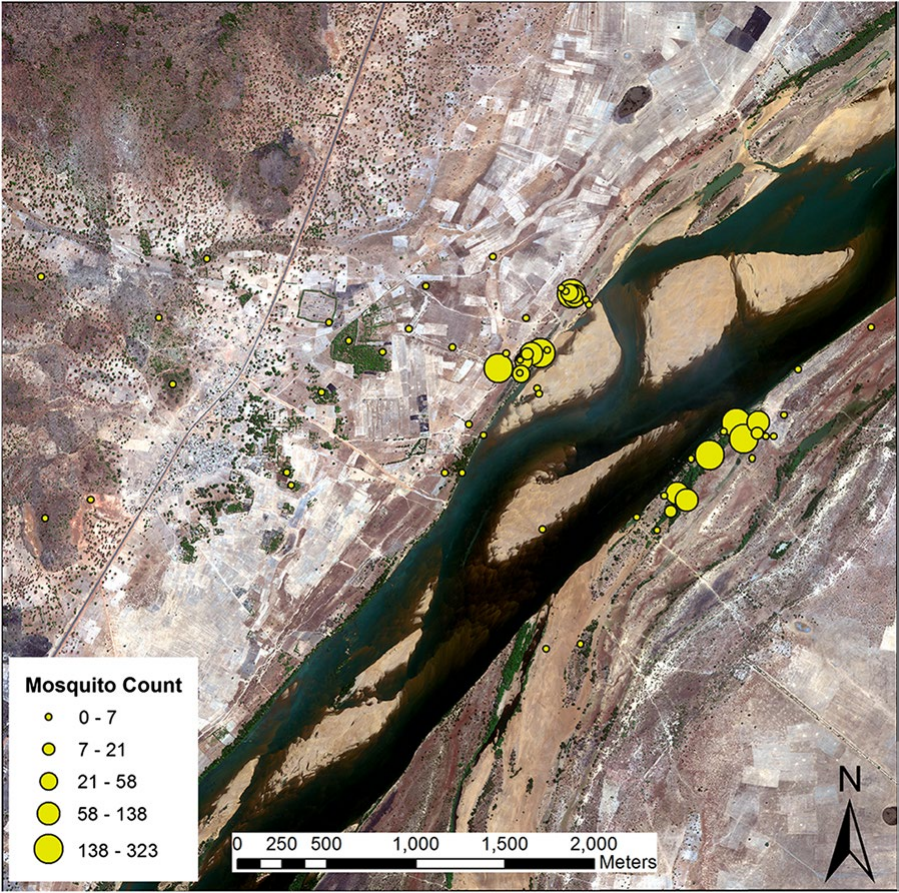
Resting catch data. The size of the *yellow circles* is scaled to reflect the number of mosquitoes caught in each location

Only *An. gambiae* mosquitoes were found in field sampling. 34 of the sampled points produced no resting *An. gambiae* mosquitoes during any point of the sampling period and at sampling locations where *An. gambiae* mosquitoes were found the number of mosquitoes captured range from a single mosquito up to 323 mosquitoes. In total, 2190 *An. gambiae* mosquitoes were collected, with 1595 females and 595 males. During morning sampling, a total of 1219 mosquitoes were collected, and 971 were obtained during the afternoons. PCR analysis of 50 adults indicated that all collected specimens belonged to *An. gambiae s.s*. No attempt was made to distinguish between *An. gambiae* and *Anopheles coluzzii,* [55]. Figure 4 provides a depiction of mosquito catches per sampling location. Further, Moran’s I produced values of 0.091, a variance of 0.008, a z-score of 1.17, and a p value of 0.24, which indicates that the spatial structure of the field data did not appear to be significantly different from random. Further, a Pearson correlation was performed between the average amount of female and male mosquitoes captured per site. The results of this analysis are shown in Table 1. A high Pearson correlation coefficient of 0.916 suggests that there was no significant difference in resting site preference between males and females.

**Fig. 5:**
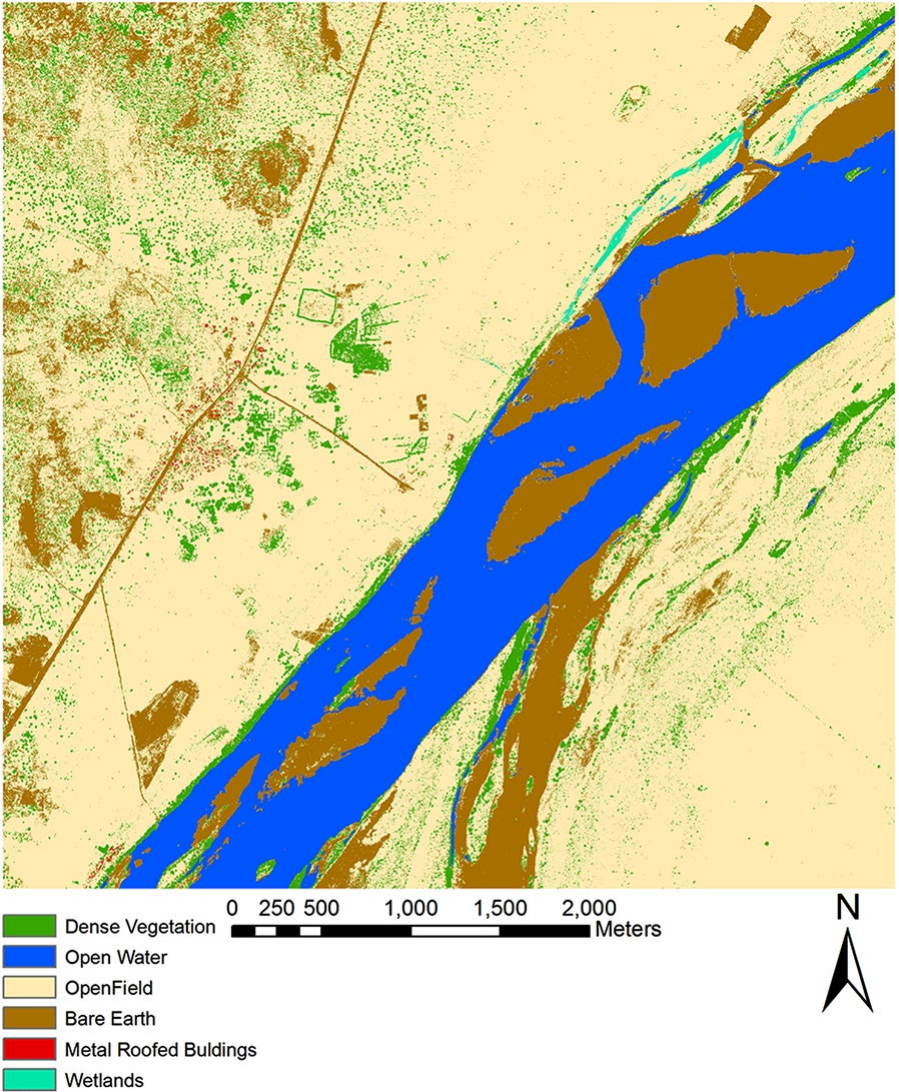
Land cover map based on WorldView 2 imagery covering the study site. The map shows the different land cover classes obtained from supervised classification and segmentation followed by manual correction to correctly delineate wetland sites

The nearest neighbour analysis of points used to validate the imagery indicated a spatially random distribution (z-score = −1.60 and a p value of 0.109) (Fig. 5). The error matrix revealed that the overall accuracy of the classified map was 82 %. Individual class accuracies related to anopheline habitats were generally high (Table 2). For example, the accuracies for dense vegetation, water, and metal-roofed buildings were of 96, 97.7, and 90.9 % respectively. In addition, the open field class had an accuracy of 95.6 %, while the bare earth class had an accuracy of 60.7 %.

**Table 2:** Classification accuracy

Class	Accuracy (%)
Dense vegetation	96
Water	97.7
Metal roofed buildings	90.9
Open field	95.6
Bare earth	60.7
Overall accuracy	82

### D-S model analysis

**Fig. 6:**
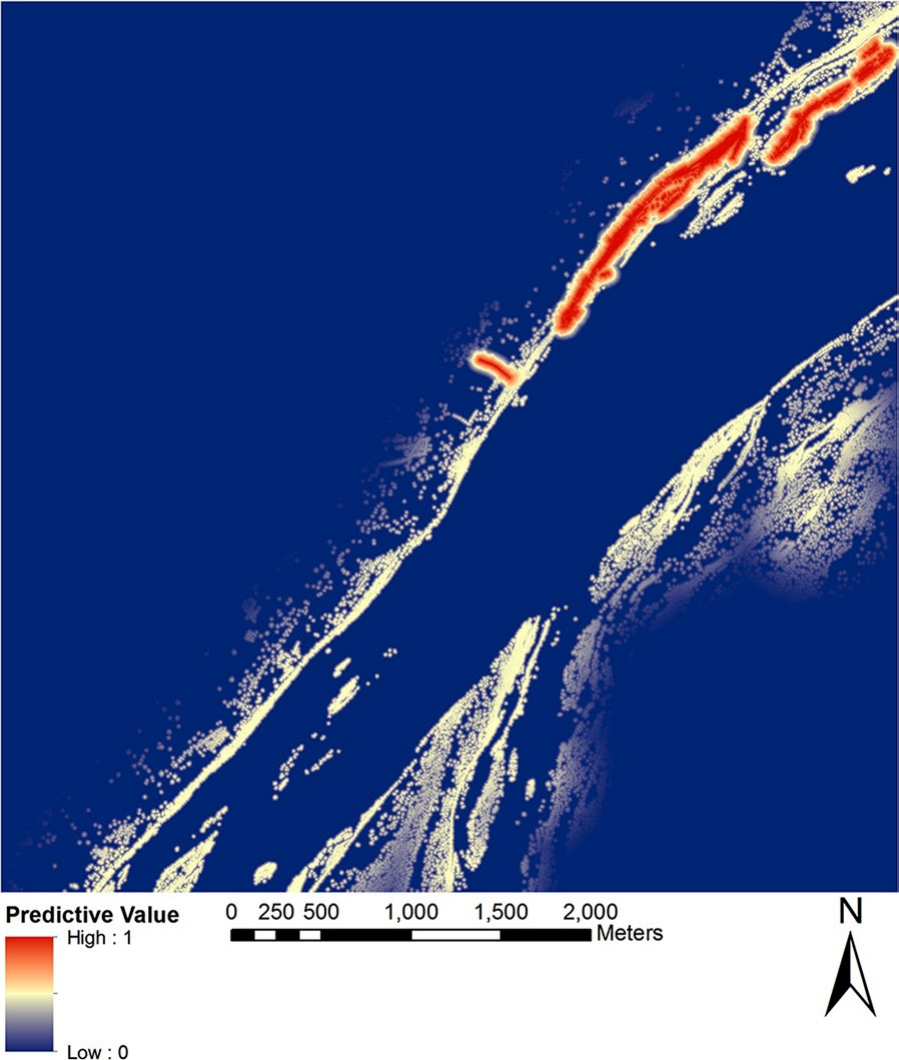
Final Dempster-Shafer (D-S) model of outdoor resting sites (belief output) for *An. gambiae*. Each pixel value provides a probability estimate of *An. gambiae* presence

**Fig. 7:**
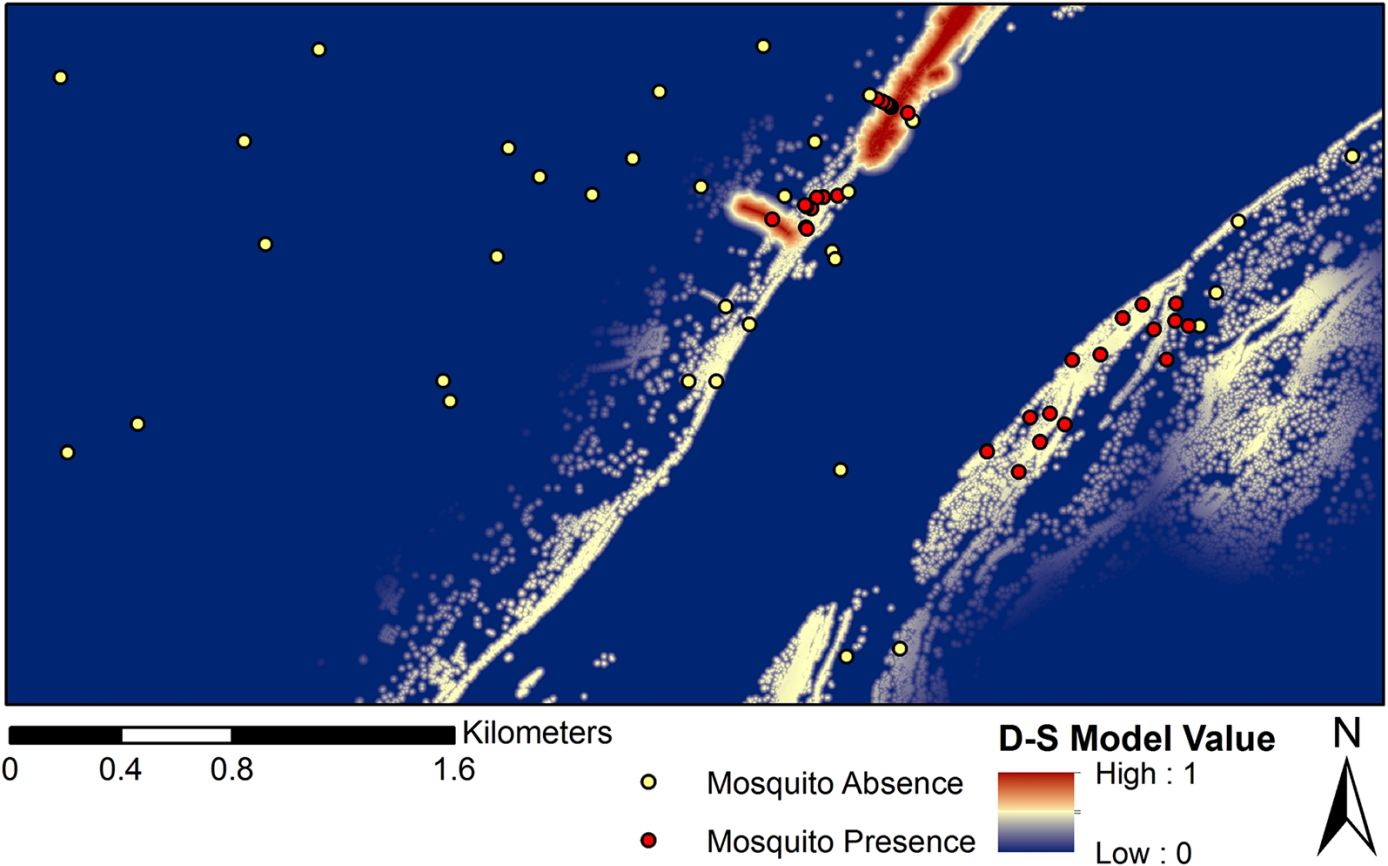
Comparision of mosquito presence/absence with the final D-S model

**Fig. 8:**
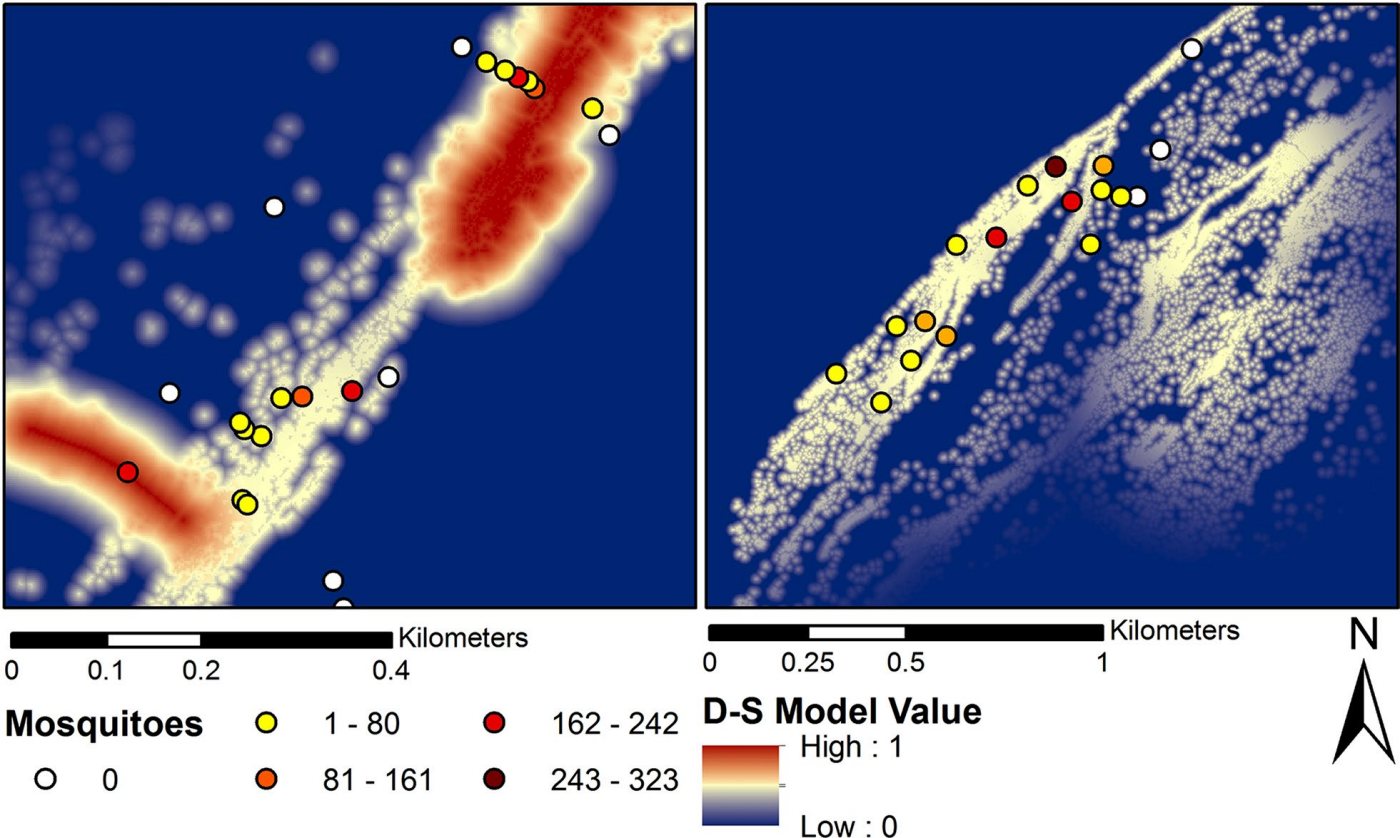
Mosquito abundances at the field sites compared to final D-S model

Figure 6 shows the D-S model belief output, which represents expected outdoor resting habitats. Meanwhile, Table 3 provides the parameters pertaining to the break points and the functions used for the individual land covers used in the D-S model creation. The model surface in Fig. 6 indicates that resting habitats were generally restricted to specific locations near the Niger River and other water bodies (wetlands and open water), particularly densely vegetated areas. The findings from the belief output are supported by previous studies indicating that shade and moisture availability are primary factors for exophilic resting habitats [22, 23, 30–32, 35]. The majority of the study site was found to be unsuitable for resting, despite possible proximity to human settlements (i.e., a proxy for blood meal availability). Direct comparisons between the D-S model output and the mosquito data are shown in Figs. 7 and 8.

**Table 3:** D-S model parameters

Parameter	Land cover feature	Variant	Function	C-breaking point (m)	D-breaking point (m)
Wetlands	Wetlands	Decreasing	Linear	0	77.5
Dense vegetation near open water*	Open water	Decreasing	Sigmoidal	0	569.75
	Dense vegetation**	Decreasing	Linear	0	17.5

Land cover feature describes the land cover that has been extracted from the classified image for the creation of the parameter. C-breaking point refers to the distance from the land cover at which the fuzzy value begins to drop from 1 down to a value of 0 with that distance indicated by breaking point D. The decreasing variant described that the fuzzy value approached 0 rather than 1. The function refers to what method of slope was used to create the fuzzy layer

* Parameter relied on two separate fuzzy layers overlaid through multiplication

** User-defined linear relationship, where the maximum predictive value was set at 0.50

**Fig. 9:**
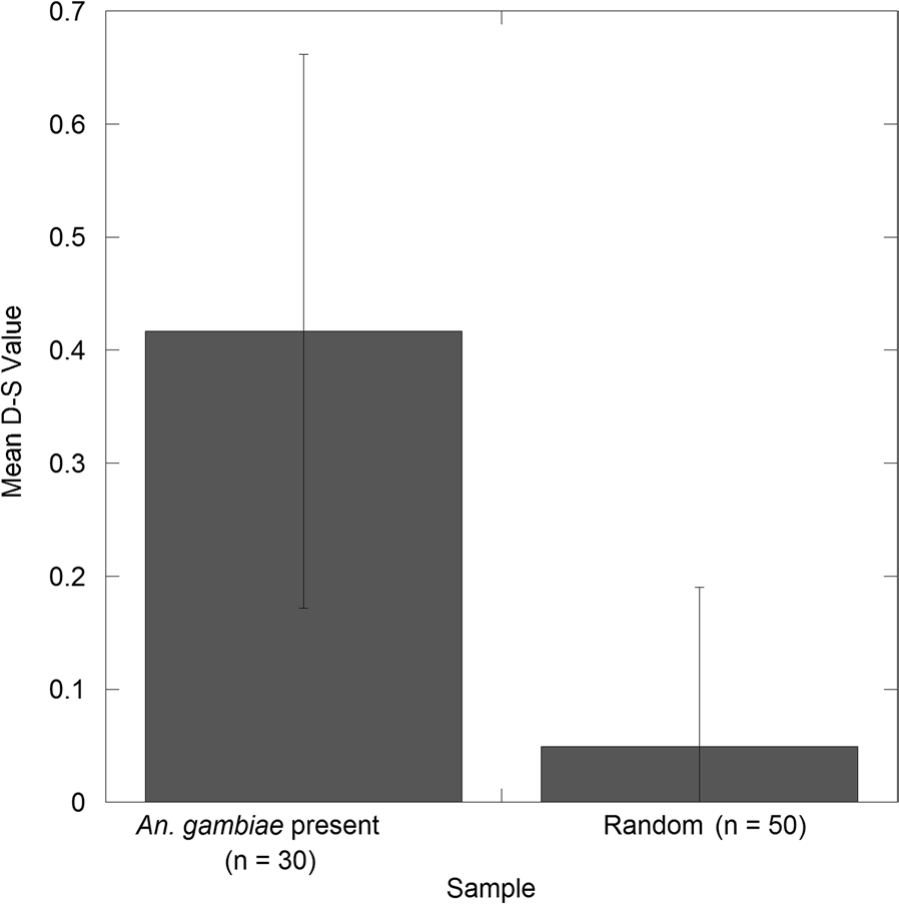
Mean of D-S values for locations where *An. gambiae* specimens were collected versus the D-S mean for 50 randomly sampled points.* Error bars* show the standard deviation

Consistent with the qualitative analysis of the model surface (Fig. 6), the *t* test comparing mean values for positive locations (n = 30) with 50 randomly sampled points indicated a significant difference (p < 0.001) between these two samples (Fig. 9). Further, the negative binomial regression failed to indicate a significant relationship (p > 0.05) between mosquito counts and the D-S surface values. However, when the same analysis was performed after removal of an outlier where a high mosquito count was obtained (n = 323 catches), the relationship between abundance and D-S model values was significant (Wald Chi Square = 18.86, p = 0.00, df = 14). This suggests that the D-S model results produced a relatively realistic depiction of both presence and abundance of *An. gambiae* at low to moderate density, but appeared insufficient to estimate areas of very high density populations. This assumption is consistent with the work of other researchers [56, 57] who have found positive relationships between predicted probability of presence and abundance within a range of taxa when using species distribution models such as, Maxent.

## Discussion

This study is the first to employ D-S modelling to assess probability of outdoor resting habitats of *An. gambiae* at a scale that is relevant for guiding vector management. Both the *t* test and a negative binomial model suggest that the D-S model performed well in distinguishing between suitable and unsuitable outdoor resting microhabitats as well as predicting the abundance of resting anopheline mosquitoes at these sites at low to moderate densities ranging from about 0–60 specimens per site. This study’s D-S model surface thus helps to advance understanding of exophilic resting ecology of *Anopheles* mosquitoes. Specifically the model indicates that shaded areas nearby water sources are important to exophilic resting habitat selection. This can be seen in the breakpoints used for the fuzzy layers of dense vegetation and open water shaded areas in Table 3. Dense vegetation surrounding open water are likely to have higher amounts of soil moisture. The optimal resting sites were found to be shaded areas close to shallow water bodies such as those represented by the wetlands class of the classified image.

While the D-S model used distances from major land cover type as a way of predicting location of resting habitats, land cover per se is largely a proxy for microclimatic conditions that favour mosquito thermoregulation. These conditions include areas of moist soil that are shaded by a dense over-story of trees and shrubs and are proximate to water sources or influenced by run-on from sheet flow. Further analysis of plant species diversity within the dense vegetation class may yield further insights on resting habitat suitability especially flowering plants that provide sugar-feeding opportunities. Additional investigation may also include assessment of leaf-area index as way to understand how variable sub-canopy light environments provided by trees and shrubs may provide suitable resting sites within clumps of woody vegetation. This study assumes that all areas covered by the dense vegetation class are spatially homogeneous in their provision of shade and sugars. However, it is likely that some tree species may provide more shade and feeding opportunities than others due to different leaf areas, and this may affect the quality of resting habitats. Furthermore, flowering phenology may control the distribution of sugar resources available at different points in the seasonal cycle and therefore the distribution and density of anophelines may shift in response to the availability of plant nectars. Thus, flowering patterns would likely impact mosquito concentrations and would likely lead to the development of more robust spatial predictions of habitat suitability during the seasonal cycle. The inability for the current D-S model to accurately take these conditions into consideration may account for the model’s limited ability to predict areas of high mosquito density. Finally, this study sampled mosquitoes in the herbaceous layer only and further sampling of resting habitats in the tree and shrub canopies may advance understanding of how resting anophelines are distributed among different canopy layers.

Despite several limitations, the D-S model driven by a small set of satellite-derived land-cover classes provided a relatively simple way to map probability of presence at high spatial resolution. Previous research for mapping mosquito presence at the village scale in Africa, [57] evaluated similar spatial patterns. A number of other studies have employed 30-m Landsat imagery [58, 59] to derive robust surfaces that indicate where transmission and breeding is likely. In contrast, this study utilized very high-resolution satellite imagery to pinpoint localized environmental factors that are likely to affect vector presence in the environment, which were transformed through the D-S modelling process to a high-resolution probability surface. Overall, however, this approach was consistent with Landsat-based approaches [57, 58] that have demonstrated a clear association between moist, alluvial locations near villages and occurrence of infective anophelines.

## Conclusion

The insights into resting habitat selection, as revealed by the D-S model provided valuable information for guiding control of anophelines at local scales, which often target specific villages and peri-urban environments where transmission remains problematic. This study also suggests that D-S modelling of resting habitats may be applicable using coarser resolution imagery such as Landsat (i.e., 30 m), which covers much larger areas relative to the WorldView 2 imagery used in our study. Landsat imagery may be used to produce classified maps that delineate land cover features, such as small waterbodies, and associated wetlands, as well as patches of dense woody vegetation, with high degrees of accuracy [60]. In this way, more spatially extensive maps may be generated to extend mapping of vector resting and sugar-feeding habitats in sub-Saharan Africa.

## Abbreviations

ATSB: attractive toxic sugar baits
D-S: Dempster Shafer
IRS: indoor residual spraying
ITNs: insecticide-treated nets
